# Expression profiling of nuclear receptors in breast cancer identifies TLX as a mediator of growth and invasion in triple-negative breast cancer

**DOI:** 10.18632/oncotarget.3942

**Published:** 2015-05-13

**Authors:** Meng-Lay Lin, Hetal Patel, Judit Remenyi, Christopher R.S. Banerji, Chun-Fui Lai, Manikandan Periyasamy, Ylenia Lombardo, Claudia Busonero, Silvia Ottaviani, Alun Passey, Philip R. Quinlan, Colin A. Purdie, Lee B. Jordan, Alastair M. Thompson, Richard S. Finn, Oscar M. Rueda, Carlos Caldas, Jesus Gil, R. Charles Coombes, Frances V. Fuller-Pace, Andrew E. Teschendorff, Laki Buluwela, Simak Ali

**Affiliations:** ^1^ Department of Surgery & Cancer, Imperial College London, London, UK; ^2^ Division of Cancer Research, University of Dundee, Ninewells Hospital & Medical School, Dundee, UK; ^3^ Statistical Genomics Group, UCL Cancer Institute, University College London, London, UK; ^4^ Centre of Mathematics and Physics in Life & Experimental Sciences, University College London, UK; ^5^ Dundee Cancer Centre, Clinical Research Centre, University of Dundee, Ninewells Hospital & Medical School, Dundee, UK; ^6^ Department of Surgical Oncology, MD Anderson Cancer Center, Houston, USA; ^7^ Geffen School of Medicine at UCLA, Los Angeles, CA USA; ^8^ Cancer Research UK Cambridge Institute, University of Cambridge Li Ka Shing Centre, Cambridge, UK; ^9^ Cell Proliferation Group, MRC Clinical Sciences Centre, Imperial College London, Hammersmith Campus, London, UK

**Keywords:** cancer, nuclear receptors, expression profiling, breast cancer, tumour classification

## Abstract

The Nuclear Receptor (NR) superfamily of transcription factors comprises 48 members, several of which have been implicated in breast cancer. Most important is estrogen receptor-α (ERα), which is a key therapeutic target. ERα action is facilitated by co-operativity with other NR and there is evidence that ERα function may be recapitulated by other NRs in ERα-negative breast cancer. In order to examine the inter-relationships between nuclear receptors, and to obtain evidence for previously unsuspected roles for any NRs, we undertook quantitative RT-PCR and bioinformatics analysis to examine their expression in breast cancer. While most NRs were expressed, bioinformatic analyses differentiated tumours into distinct prognostic groups that were validated by analyzing public microarray data sets. Although ERα and progesterone receptor were dominant in distinguishing prognostic groups, other NR strengthened these groups. Clustering analysis identified several family members with potential importance in breast cancer. Specifically, RORγ is identified as being co-expressed with ERα, whilst several NRs are preferentially expressed in ERα-negative disease, with TLX expression being prognostic in this subtype. Functional studies demonstrated the importance of TLX in regulating growth and invasion in ERα-negative breast cancer cells.

## INTRODUCTION

Breast cancer is the most common cancer diagnosed in women, making up 23% of all cancers in women, with 1.38 million new cases worldwide annually and is responsible for 460,000 deaths [1]. The hormone estrogen is a key proliferative driver in breast cancer and acts by binding to estrogen receptor-α (ERα), resulting in its activation. Therefore, breast cancer patients are stratified on the basis of tumour expression of ERα. Inhibition of ERα activity using the antagonist tamoxifen, or drugs that inhibit estrogen biosynthesis by blocking the activity of aromatase, a key enzyme in estrogen biosynthesis (e.g. anastrozole, letrozole) [2], provide important strategies for the management of ERα-positive breast cancer and significantly reduce recurrence and breast cancer mortality.

Despite the benefits of endocrine agents for many patients, a large proportion of patients with ERα-positive breast cancer progress on anti-estrogen or aromatase inhibitor therapies, or relapse following initial response [3, 4]. Moreover, current hormonal agents are ineffective for a fifth of breast cancers that do not express ERα. These problems highlight the need for improvements in the understanding of ERα-positive breast cancer, to develop additional markers that will identify those patients who will respond to hormone therapies, for developing new therapies for ERα-positive patients who do not respond to current endocrine therapies and for identifying new agents to treat patients with ERα-negative disease.

Nuclear receptors (NR) are typically activated upon binding to small molecules, including steroid hormones, retinoids, lipids and xenobiotics and play critical roles in growth, development, tissue homeostasis and metabolism [5]. Consequently, deregulation of NR action is important in many diseases, including cancer. Regulation of NR activity by small molecule ligands has facilitated the development of drugs that mimic the function of cognate ligands (agonists) or act as inhibitors (antagonists), a process that is further facilitated by the availability of ligand binding domain crystal structures for most NRs. Examples of antagonists as cancer therapeutics include inhibitors of estrogen (tamoxifen, fulvestrant), androgen (flutamide, bicalutamide, MDV3100) and progesterone (mifepristone, onapristone, lonaprisan) receptors. Therapeutic synthetic agonists and antagonists, many being approved for clinical use, have also been developed for many other NRs, including the glucocorticoid (GR; dexamethasone), vitamin D3 (VDR), retinoid, and peroxisome proliferator activated (PPARs) receptors [6]. Therefore, identification of NRs with functional roles in breast cancer development and progression has the potential for rapid progression to the clinic.

The progesterone receptor (PGR) is well-established as an ERα-regulated gene Indeed, clinical practice includes the routine immunohistochemical determination of PGR; PGR expression is almost always observed only in ERα positive breast cancer [7] and its presence is a presumed marker of ERα functionality. Furthermore, increased risk of breast cancer has been reported for post-menopausal women with hormone replacement therapies (HRT) that include synthetic progestins [8]. Hence, anti-progestins have been tested in the metastatic setting [9] and clinical trials using the anti-progestin mifepristone in the early stage breast cancer setting are underway (ClinicalTrials.gov: NCT01138553).

Importantly, recent studies provide new evidence to show that some NRs act co-operatively with ERα in breast cancer cells, often through co-regulation of gene expression. Thus, retinoic acid receptor-α (RARα), whose expression is estrogen-regulated in breast cancer, localizes to ERα binding sites to modulate the expression of ERα target genes [10, 11], establishing crosstalk between estrogen and retinoid signalling that is important for the growth of ERα-positive breast cancer cells. LRH-1 is another ERα-regulated NR, which stimulates proliferation and promotes motility and invasion of breast cancer cells and regulates the expression of estrogen-responsive genes in ERα+ breast cancer cells, acting in a co-operative manner with ERα [12].

The androgen receptor (AR), another NR whose expression is strongly associated with ERα expression in breast cancer [13], inhibits expression of estrogen responsive genes in ERα-positive breast cancer cells. Remarkably, in a small subset of ERα-negative “molecular apocrine” breast cancer [14], AR activates, rather than inhibits, the expression of many ERα target genes through its recruitment to sites that are normally bound by ERα in luminal MCF7 cells [15], suggesting that in ERα-negative breast cancer some NRs can, at least in part, take the role of ERα.

It appears, therefore, that NRs co-expressed with ERα play important roles in the regulation of gene expression by ERα and consequently in breast cancer, whilst other NRs have been implicated in ERα-negative breast cancer [16, 17]. These findings prompted us to determine the expression profiles of all NRs in different breast cancer subtypes using quantitative RT-PCR (qRT-PCR). Our analysis confirms the co-expression of several NRs with ERα, but also identifies other NRs whose expression is strongly associated with ERα, in particular a previously unreported relationship with retinoic acid receptor-related orphan receptor-γ (RORγ). Further, we show that expression of the Tailless homolog TLX (NR2E1) is negatively associated with ERα, TLX being expressed in ERα-negative breast cancer and confirm this relationship through analysis of published microarray data sets. Functional studies demonstrate that TLX regulates breast cancer cell growth and invasion, identifying TLX as a new therapeutic target in breast cancer.

## RESULTS

### Quantitative RT-PCR profiling demonstrates expression of the majority of nuclear receptors in breast cancer

**Figure 1:**
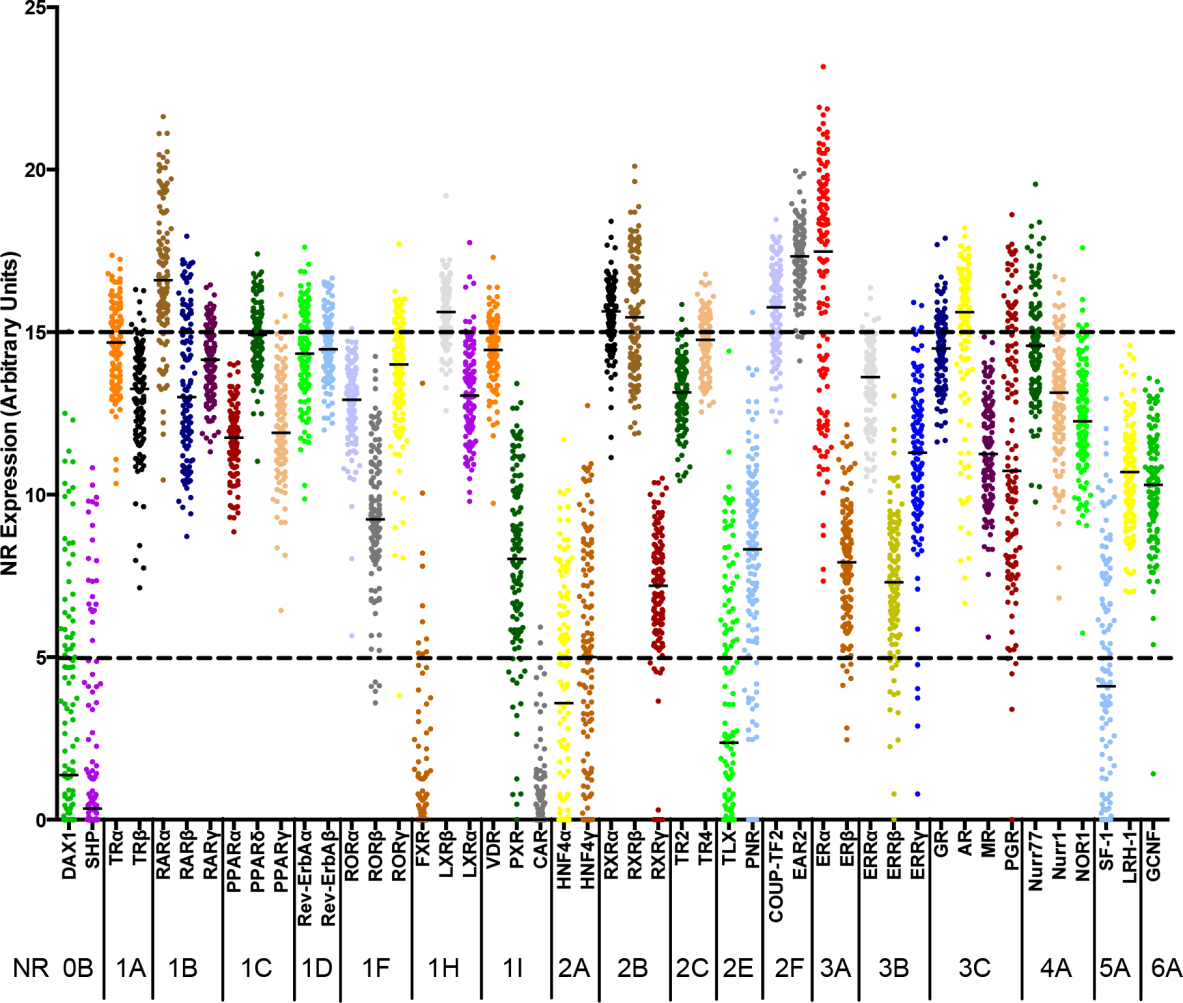
Relative expression of Nuclear Receptors in RNA prepared from breast tumours The normalized mRNA expression of each NR is shown for RNAs prepared from 128 breast tumours. Each dot represents one patient sample. Real-time RT-PCR was performed for all NR, excepting COUP-TF1. The NRs are ordered using the NR superfamily nomenclature: 0B, DAX-like receptors; 1A, Thyroid Hormone Receptors; 1B, Retinoic acid receptors; 1C, Peroxisome proliferator-activated receptors; 1D, Rev-Erb receptors; 1F, RAR-related orphan receptors; 1H, Liver X receptor-like receptors; 1I, Vitamin D receptor-like receptors; 2A, Hepatocyte nuclear factor-4 receptors; 2B, Retinoid X receptors; 2C, Testicular receptors; 2E, Tailless-like receptors; 2F, COUP-TF-like receptors; 3A, Estrogen receptors; 3B, Estrogen-related receptors; 3C, 3-Ketosteroid receptors; 4A, Nerve growth factor IB-like receptors; 5A, Fushi tarazu F1-like receptors; 6A, Germ cell nuclear factor receptors.

To determine the NR expression profiles in tumours representative of different breast cancer subtypes, total RNA was prepared from 128 breast tumours from the Tayside Tissue Bank, for which clinical and histopathological details were available, as well as long term clinical follow-up. For qRT-PCR, we designed a Taqman TLDA card, to include assays for 47 NR, together with GAPDH as a control. This arrangement excluded an assay for COUP-TF1 (NR2F1), since the only real-time assays available for COUP-TF1 at the time also detect COUP-TF2 (NR2F2). Most NRs were expressed in this patient cohort, with ERα, RARα, EAR2, COUP-TF2 and RXRβ being the most highly expressed NRs (Figure 1). Interestingly, COUP-TF2 and EAR2 expression levels were high in ERα-positive, as well as ERα-negative tumours (Table 1; Supplementary Figures 1–2). Although more highly expressed in ERα-positive breast cancer, high-level RARα expression was also evident in ERα-negative tumours. Expression of CAR, SHP and FXR was undetectable in the great majority of cases.

**Table 1:** Expression levels of nuclear receptors in 128 breast cancers

High expression		Moderate expression (15 > to > 5)				Low/Absent expression (≤ 5)	
**All Tumors**							
**ERα**	**17.49** (13.55 – 19.36)	**PPARδ**	**14.92** (14.24 – 15.45)	**RORα**	12.93 (12.19 – 13.57)	**SF-1**	4.11 (0.73 – 7.28)
**EAR2**	**17.34** (16.77 – 17.93)	**TR4**	**14.77** (14.00 – 15.42)	**NOR1**	12.26 (11.33 – 13.37)	**HNF4α**	3.59 (−0.10 – 6.65)
**RARα**	**16.61** (15.58 – 18.06)	**TRα**	**14.69** (13.82 – 15.52)	**PPARγ**	11.91 (11.22 – 13.06)	**TLX**	2.38 (0.02 – 6.21)
**COUP-TF2**	**15.77** (14.85 – 16.62)	**Nurr77**	**14.59** (13.49 – 15.57)	**PPARα**	11.75 (11.20 – 12.34)	**DAX1**	1.38 (−0.58 – 5.24)
**RXRα**	**15.65** (15.01 – 16.27)	**GR**	**14.51** (13.67 – 15.24)	**ERRγ**	11.30 (9.83 – 12.58)	**SHP**	0.34 (−0.77 – 3.42)
**LXRβ**	**15.62** (15.10 – 16.21)	**Rev-ErbAβ**	14.48 (13.96 – 15.19)	**MR**	11.26 (10.13 – 12.6)	**FXR**	−0.07 (−1.06 – 1.33)
**AR**	**15.62** (14.19 – 16.36)	**VDR**	14.46 (13.99 – 15.00)	**PGR**	10.74 (8.10 – 15.15)	**CAR**	−0.11 (−1.07 – 0.78)
**RXRβ**	**15.46** (14.01 – 17.00)	**Rev-ErbAα**	14.35 (13.52 – 15.39)	**LRH-1**	10.70 (9.41 – 11.63)		
		**RARγ**	14.15 (13.34 – 14.90)	**GCNF**	10.31 (9.33 – 11.39)		
		**RORγ**	14.01 (12.84 – 14.95)	**RORβ**	9.25 (8.22 – 10.75)		
		**ERRα**	13.62 (12.33 – 14.28)	**PNR**	8.33 (6.30 – 10.05)		
		**TRβ**	13.26 (12.11 – 14.30)	**PXR**	8.03 (6.24 – 10.13)		
		**TR2**	13.15 (12.53 – 13.81)	**ERβ**	7.93 (6.66 – 8.94)		
		**NURR1**	13.14 (11.91 – 14.00)	**ERRβ**	7.31 (6.28 – 8.71)		
		**LXRα**	13.05 (12.06 – 14.09)	**RXRγ**	7.20 (6.01 – 8.24)		
		**RARβ**	13.00 (11.71 – 15.30)	**HNF4γ**	5.01 (1.39 – 7.77)		
**ERα-positive**							
**ERα**	18.7 (17.24 – 19.95)	**PPARδ**	14.93 (14.27 – 15.48)	**RORα**	13.02 (12.50 – 13.71)	**HNF4γ**	4.97 (1.43 – 8.16)
**EAR2**	17.52 (17.03 – 18.03)	**TRα**	14.90 (14.29 – 15.74)	**RARβ**	12.91 (11.54 – 15.21)	**HNF4α**	4.11 (0.24 – 6.84)
**RARα**	17.24 (15.91 – 18.84)	**TR4**	14.86 (14.06 – 15.48)	**NOR1**	12.40 (11.51 – 13.49)	**SF-1**	3.57 (0.55 – 6.65)
**LXRβ**	15.95 (15.29 – 16.43)	**Nurr77**	14.78 (13.97 – 16.27)	**PPARγ**	12.10 (11.40 – 13.10)	**TLX**	0.86 (−0.51 – 3.56(Continued ))
**COUP-TF2**	15.93 (15.11 – 16.78)	**Rev-ErbAβ**	14.72 (14.06 – 15.42)	**PPARα**	11.52 (11.07 – 12.07)	**DAX1**	0.69 (−0.67 – 4.61)
**AR**	15.88 (15.15 – 16.56)	**GR**	14.60 (13.98 – 15.33)	**ESRRG**	11.31 (10.25 – 12.38)	**SHP**	0.56 (−0.67 – 3.8)
**RXRα**	15.87 (15.37 – 16.35)	**VDR**	14.58 (14.08 – 15.00)	**MR**	11.26 (10.17 – 12.65)	**FXR**	0.1 (−0.75 – 1.31)
**RXRβ**	15.37 (14.10 – 17.44)	**Rev-ErbAα**	14.51 (13.90 – 15.63)	**LRH-1**	11.01 (9.83 – 11.74)	**CAR**	0.1 (−0.86 – 0.79)
		**RARγ**	14.46 (13.79 – 15.13)	**GCNF**	10.40 (9.39 – 11.67)		
		**RORγ**	14.35 (13.45 – 15.08)	**RORβ**	9.75 (8.81 – 11.46)		
		**PGR**	13.98 (10.34 – 15.81)	**PNR**	9.38 (7.87 – 10.60)		
		**TRβ**	13.61 (12.45 – 14.42)	**ERβ**	7.90 (6.55 – 8.87)		
		**ERα**	13.49 (12.33 – 14.27)	**PXR**	7.88 (6.30 – 10.19)		
		**NURR1**	13.34 (12.40 – 14.21)	**ERRβ**	7.86 (6.81 – 8.97)		
		**TR2**	13.27 (12.82 – 14.02)	**RXRγ**	7.60 (6.19 – 8.66)		
		**LXRα**	13.10 (12.12 – 14.07)				
**ERα-negative**							
**EAR2**	16.89 (16.15 – 17.46)	**PPARδ**	14.91 (14.2 – 15.34)	**ERα**	12.14 (11.18 – 13.55)	**DAX1**	4.54 (−0.08 – 6.06)
**RARα**	15.54 (13.79 – 16.64)	**TR4**	14.59 (13.91 – 14.92)	**NURR1**	12.12 (11.12 – 13.36)	**HNF4α**	2.36 (−0.43 – 5.82)
**RXRβ**	15.49 (13.89 – 16.51)	**VDR**	14.29 (13.41 – 14.93)	**NOR1**	11.89 (11.27 – 13.14)	**SHP**	−0.18 (−1.35 – 2.63)
**COUP-TF2**	15.37 (14.22 – 15.96)	**GR**	14.23 (13.28 – 14.83)	**PPARG**	11.50 (10.94 – 12.96)	**FXR**	−0.27 (−1.73 – 1.26)
**LXRβ**	15.29 (14.72 – 15.73)	**Rev-ErbAβ**	14.15 (13.53 – 14.56)	**MR**	11.25 (10.05 – 12.46)	**CAR**	−0.27 (−1.67 – 0.64)
**RXRα**	14.99 (14.37 – 15.53)	**TRα**	14.06 (13.08 – 15.15)	**ERRγ**	10.87 (9.12 – 13.03)		
		**Nurr77**	13.88 (13.16 – 14.65)	**LRH-1**	9.94 (8.80 – 10.96)		
		**Rev-ErbAα**	13.81 (13.26 – 14.63)	**GCNF**	9.72 (8.76 – 10.85)		
		**ERRα**	13.70 (12.92 – 14.41)	**PXR**	8.49 (6.21 – 9.84(Continued ))		
		**AR**	13.68 (10.70 – 15.70)	**RORβ**	8.26 (7.08 – 9.37)		
		**RARγ**	13.58 (13.02 – 14.10)	**PGR**	8.06 (7.05 – 9.69)		
		**RARβ**	13.47 (12.31 – 15.41)	**ERβ**	8.01 (6.78 – 9.17)		
		**LXRα**	13.03 (11.99 – 14.24)	**RXRγ**	6.40 (5.76 – 7.21)		
		**RORγ**	12.98 (12.32 – 14.20)	**TLX**	6.29 (3.80 – 8.40)		
		**TR2**	12.61 (11.83 – 13.47)	**ERRβ**	6.14 (5.14 – 7.35)		
		**TRβ**	12.55 (11.10 – 13.40)	**PNR**	5.41 (3.12 – 7.34)		
		**RORα**	12.50 (11.78 – 13.33)	**SF-1**	5.03 (2.32 – 7.48)		
		**PPARα**	12.26 (11.72 – 12.75)	**HNF4γ**	5.03 (1.15 – 7.28)		

Expression levels (ΔCt values) relative to undetectable expression (Ct > 35). Median expression is shown, together with the 25 and 75 percentile range.

### Consensus cluster analysis to differentiate breast cancers in different groups

**Figure 2:**
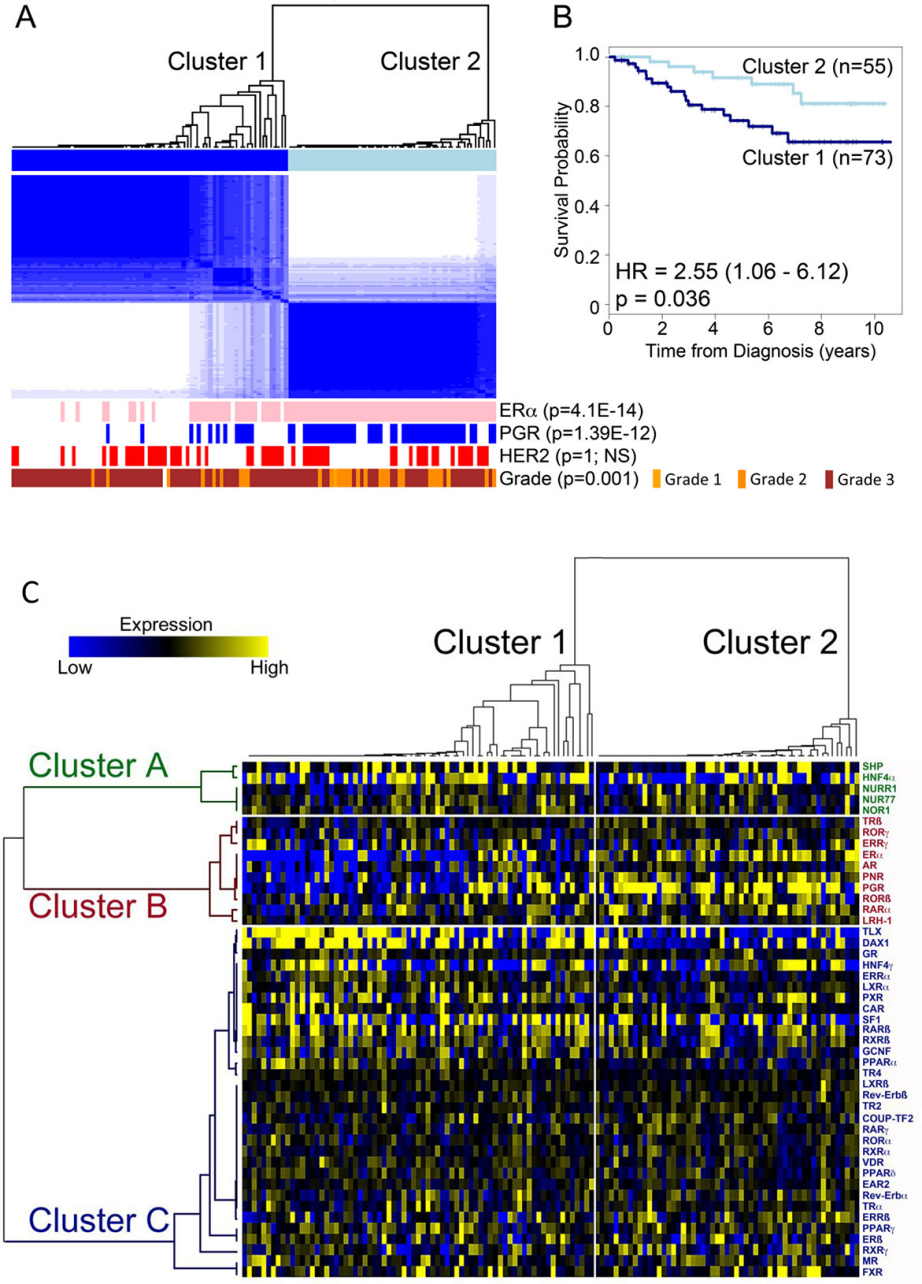
Identification of two main breast cancer subtypes based on NR gene expression **A.** Unsupervised hierarchical consensus clustering segregates tumours into two clusters based on qRT-PCR for NR expression. Shown is the ERα, PGR and HER2 IHC status for each tumour, positive tumours being depicted as pink (ERα), blue (PGR) and red (HER2) bars. Statistical significance was established using Fisher's exact test. **B.** Kaplan-Meier plot shows patient survival for the patients in the two clusters. Crosses show censored samples. **C.** Heatmap representation of hierarchical consensus cluster analysis of nuclear receptor mRNA expression. Tumours are grouped according to the two clusters identified from consensus clustering. Median centred NR expression in patient samples is shown in a heat map following the arrangement of NRs and tumour samples according to the clusters identified in the consensus cluster analysis.

Unsupervised hierarchical consensus clustering [18] was used to discern molecular subclasses of breast tumours with similar NR expression profiles. Consensus cluster analysis provided evidence for separation of the tumours into two, three or four clusters (Supplementary Figure 3), with two clusters exhibiting the most stable configuration (Figure 2A). For the two clusters, Kaplan-Meier (KM) survival plots showed that patients segregating to cluster 2 have a significantly better prognosis, compared with patients in cluster 1 (HR = 2.55 (1.06–6.12), *p* = 0.029) (Figure 2B). Determination of the association of the two clusters with clinical features showed that cluster 2 samples were likely to be of lower tumour grade (*p* = 0.001), although tumour grade trended towards, but did not reach significant association with survival in this patient cohort (Supplementary Figure 4). Moreover, there was an association between the clusters and immunohistochemically (IHC) determined ERα (*p* = 4.1E-14) and PGR (*p* = 1.4E-12) status (Figure 2A), with the better prognosis group (cluster 2) being enriched in ERα and PGR positive tumours. There was no relationship between the clusters and HER2 status (*p* = 1.0) and although patients with PGR positive disease had better survival than PGR negative patients, this also did not reach significance (*p* = 0.113) in our patient cohort (Supplementary Figure 5). Patients with IHC determined ERα-positivity had a better prognosis than the ERα-negative patients (HR = 0.41 (0.18 – 0.91); *p* = 0.024).

We have seen that ERα and PGR mRNA levels were not associated with patient outcome in univariate analysis, regardless of the expression level cut-offs used, suggesting that other NRs are important contributors to the survival differences observed for the two clusters, at least when examining expression at the mRNA level. To investigate this, we used unsupervised consensus clustering analysis with different partitions, *k* = 2 to *k* = 6, in order to cluster NR gene expression (Supplementary Figure 5). The empirical cumulative distribution function indicated an approximate best stability for *k* = 3, indicating that the optimal number of robust NR clusters in this data set is three (Figure 2C). Gene Cluster B, which contained ERα, also contained the known ERα-regulated genes PGR, RARα and LRH-1. This cluster also contained AR and PNR, both previously shown to be co-expressed with ERα in breast cancer [13, 19], as well as ERRγ. High-level ERRγ expression has previously been associated with ERα and PGR-positivity, although the statistical significance of the previously reported associations was weak (*p* = 0.054 and *p* = 0.045, respectively) [20]. Also present in the ERα cluster were TRβ, RORβ, and RORγ. The Kruskal-Wallis test, used to determine association between ERα, PGR and HER2 IHC status and PGR (*p* = 1.10E-14), AR (*p* = 8.86E-07), RARα (*p* = 7.07E-06), LRH-1 (*p* = 1.44E-03), as well as TRβ (*p* = 3.06E-04), RORβ (*p* = 2.70E-03) and RORγ (*p* = 1.18E-04) mRNA expression provides further evidence in support of the association between expression of these receptors and ERα (Supplementary Figures 1–2). The Mann-Whitney test further confirmed association between the expression of these NRs and ERα (Table 2). It should be noted that despite the co-clustering of ERRγ with ERα, the association for ERRγ did not reach significance (*p* = 0.603).

**Table 2:** Association between ERα and Select NR expression (Mann-Whitney)

Positive Association with ERα				
	qRT-PCR	METABRIC*	TCGA^#^	GSE20685^§^
Sample No. Analysed	*n* = 128	*n* = 1,980	*n* = 485	*n* = 327
ERα	3.6E-18	2.4E-188	2.8E-47	9.6E-48
PGR	4.6E-11	9.6E-102	1.7E-29	6.4E-34
PNR	1.9E-10	4.9E-110	1.8E-34	1.5E-11
	AR	7.6E-60	7.8E-28	8.0E-16
RORβ	1.9E-06	1.7E-06	1.2E-02	3.4E-01
RARα	1.3E-06	3.0E-88	1.2E-24	1.3E-07
TRβ	9.2E-05	8.1E-01	1.7E-08	1.4E-08
RORγ	2.6E-04	2.3E-44	1.5E-15	7.3E-08
LRH-1	1.9E-03	4.1E-03	5.8E-04	2.9E-01
ERRγ	6.0E-01	1.8E-05	4.4E-02	3.1E-01
**Negative Association with ERα**				
	qRT-PCR	METABRIC	TCGA	GSE20685
TLX	5.5E-07	2.7E-10	1.6E-15	1.3E-02
PPARα	3.3E-04	1.1E-34	4.3E-28	1.4E-12
DAX1	2.9E-02	1.7E-02	4.0E-03	6.7E-01

* 21 samples were excluded because of lack of follow-up information

# 51 samples were removed from analysis due to incomplete information or sample duplication

§ ERα status is based on cut-offs for ERα positivity determined from the microarray expression profiling data by the authors [44]. For the other data sets IHC determined ERα status has been applied for the analysis.

To obtain further evidence for the association between ERα expression and expression of these NRs, we analysed a number of gene expression microarray data sets. The data sets used were chosen on the basis of patient number and/or availability of follow-up information on outcome and included the METABRIC series of nearly 2, 000 cases [21], the TCGA series of about 500 cases [22] and the 300+ cases in GSE20685 [23]. As expected, there were highly statistically significant positive associations between ERα IHC status and ERα mRNA levels, as well as with PGR, AR and RARα (Table 2). Of the other NRs co-clustering with ERα in our qRT-PCR samples, evidence for co-expression of ERα with TRβ, ERRγ, RORβ or LRH-1 was less equivocal, not reaching statistical significance in one out of the three microarray datasets. However, association between ERα and PNR expression was evident, as previously reported [19]. The association between ERα and RORγ, which has not previously been described, was confirmed in the microarray series. Expression of RORγ in breast cancer cells was determined by expression analysis in breast cancer cell lines (Figure 6A), which also showed association of RORγ with ERα expression (Mann-Whitney; *p* = 0.014).

### Identification of nuclear receptors for distinguishing patient groups

**Figure 3:**
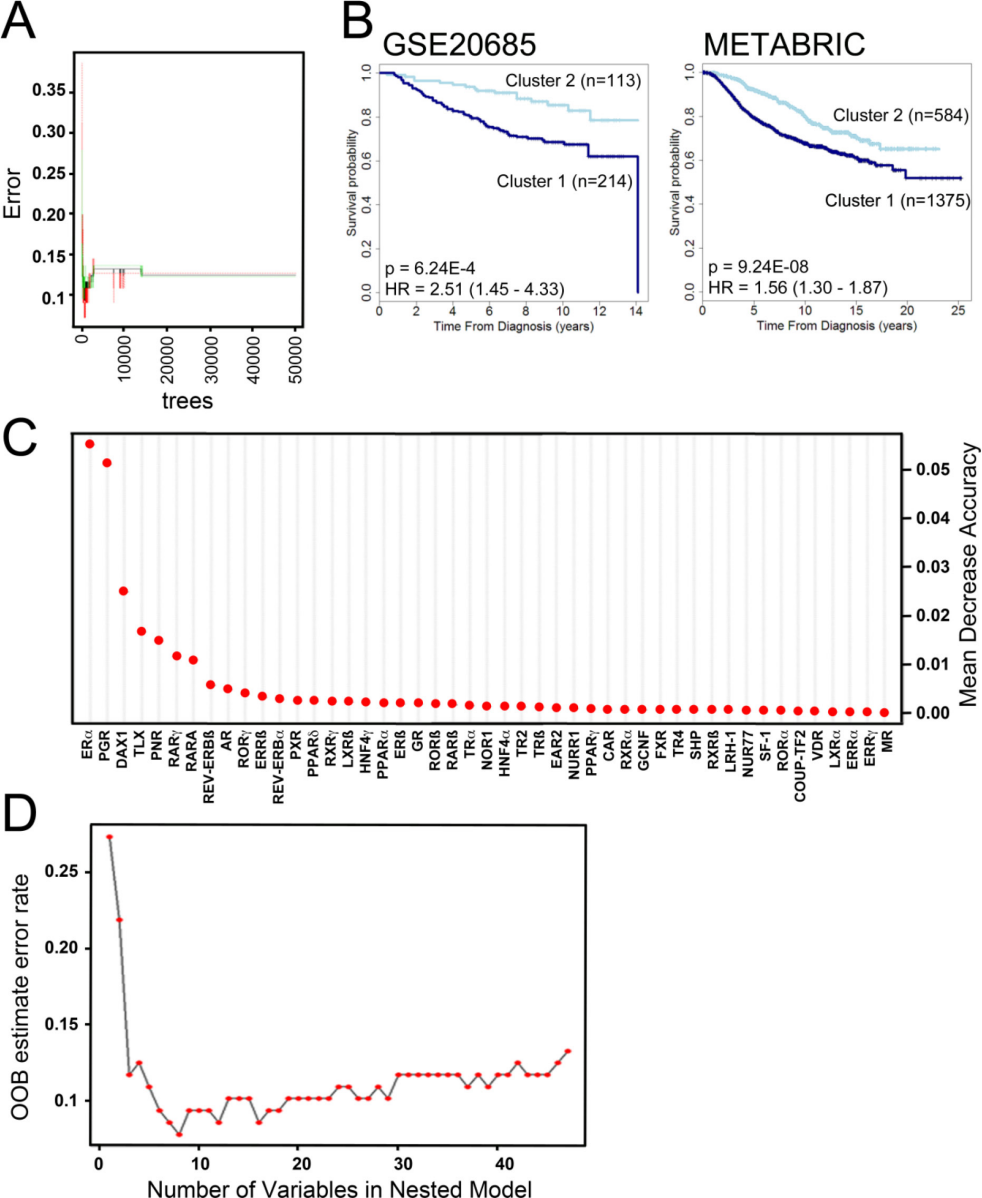
Random Forest (RF) analysis to generate a classification model for risk groups identifies NR of highest importance in generating breast cancer clusters **A.** Shown is an error rate plot for 50,000 trees generated. The curves represent the error rates for cluster 1 (red), cluster 2 (green) and the “Out-of-Bag (OOB) error rate (black). **B.** Kaplan-Meier plot shows association of the NR expression signature identified by RF analysis with disease specific survival for the GSE20685 (26) and METABRIC (28) microarray data sets. **C.** Dot chart of NR importance as measured by RF analysis. **D.** Shown is OOB error for 50,000 RF trees. The red dots represent the OOB errors for the number of NRs using in RF, each analysis excluding the least important NR. Thus, in the case of 8 NR, which yields the lowest error, only the top 8 NR from part B were used for RF.

We next determined if the consensus clustering signature generated from the qRT-PCR analysis that separates our patient cohort into good and poor prognosis groups, could be extended to the microarray data sets. To do this, we performed Random Forest (RF) classification [24] with the tumour cluster classification obtained from consensus clustering as categorical factors to generate a model using z-score transformed qRT-PCR data set. As the number of trees may affect classification error, RF analysis was carried out for 5000, 10000, 20000 and 50000 trees, with the best error stability being exhibited for 50,000 trees (Figure 3A). We then validated this classifier with two data sets, GSE20685 [23], which included patient survival information for 327 samples and the METABRIC samples (*n* = 1,959). In both cases, cluster 2 was significantly associated with better prognosis (HR = 2.32 (1.36–3.95), *p* = 0.002 and HR = 1.86 (1.51–12.30), *p* = 5.1 × 10^−9^, respectively) (Figure 3B). This indicates that the NR expression signature derived from our qRT-PCR analysis separates breast tumours into two prognostically different groups, although given the significantly greater number of ERα and PGR positive tumours in cluster 2, it is likely that ERα and PGR are important determinants in this expression signature. Indeed, plotting variable importance showed that ERα and PGR are especially important, but also highlighted several other NRs as important classifiers of clusters 1 and 2 (Figure 3C). To further confirm the important variables, RF was run re-iteratively for NRs, removing the bottom most NR (assigned the least variable importance score), one at a time. This showed that the top 8 NR, namely ERα, PGR, DAX1, TLX, PNR, RARγ, RARα and Rev-erbAβ provide the lowest error rate (Figure 3D), highlighting these NRs as the most important variables for differentiating the two tumour clusters.

**Figure 4:**
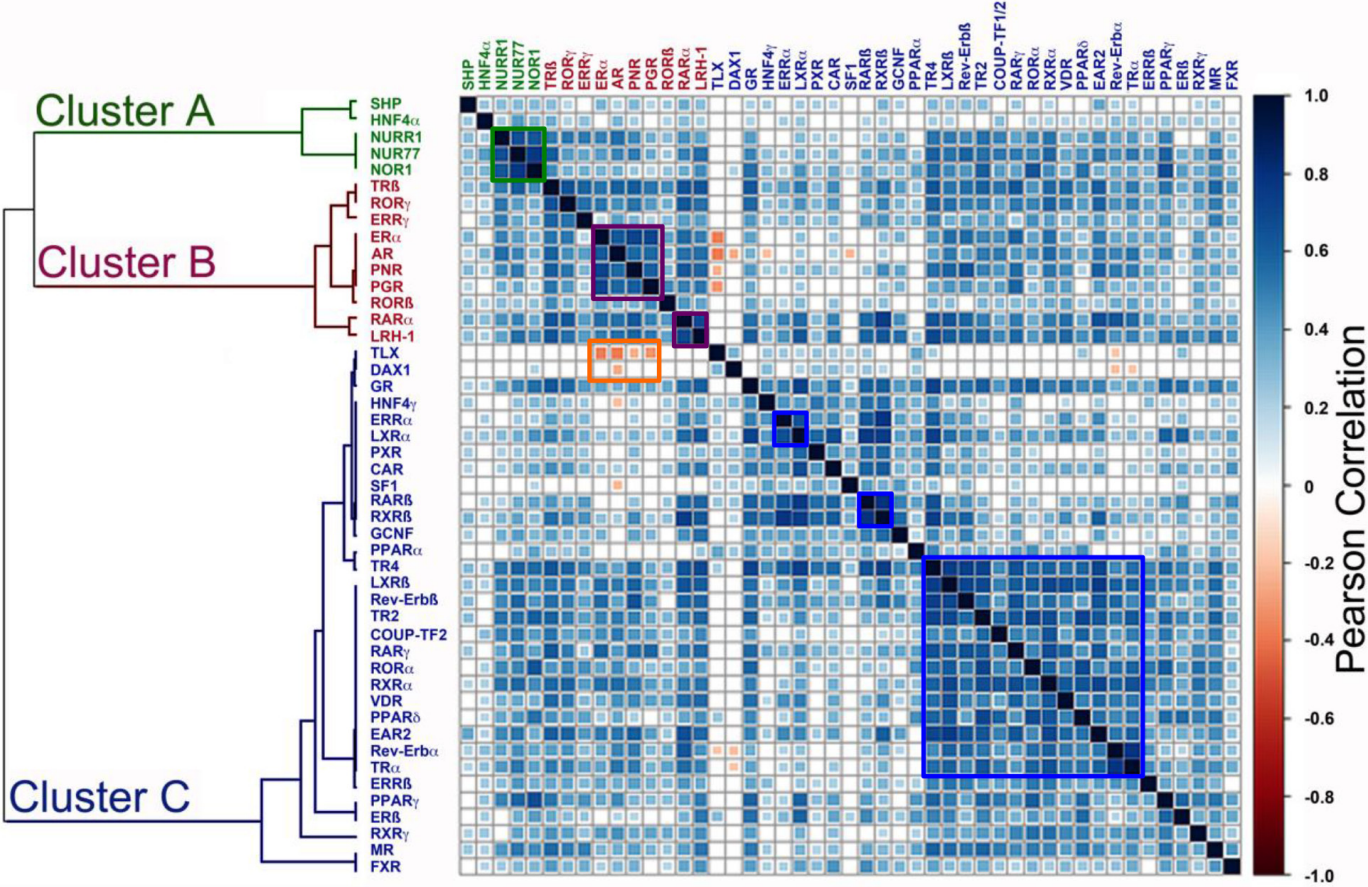
Pairwise Pearson Correlation Coefficient (PCC) analysis identifies NRs whose expression is positively or negatively associated with ERα expression **A.** Positive correlations (of 1 for self-comparisons) are depicted in blue, colour intensity reflecting the value of the correlation. The highest positive correlation value was 0.79. Negative associations are shown in red, with the colour intensity increasing with greater negative correlation, the greatest negative value being 0.37.

**Figure 5:**
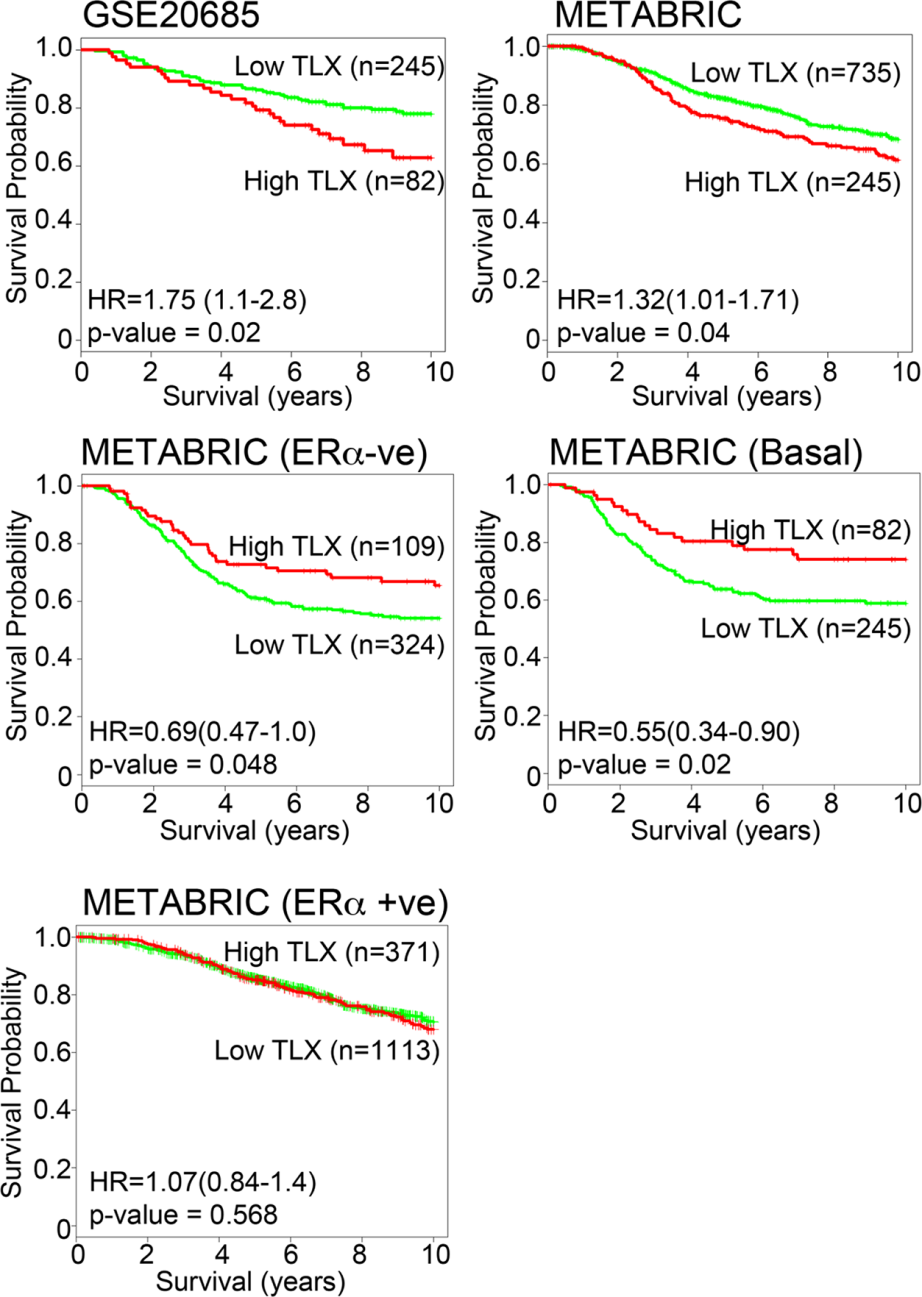
TLX expression is a marker of poor prognosis in breast cancer, but is associated with better prognosis in ERα-negative breast cancer Kaplan-Meier survival plots for TLX expression in GSE20685 [23] and METABRIC [21] microarrays is shown for all tumours and for ERα-negative and basal subtype tumours.

We next used Pairwise Pearson correlation coefficient (PCC) to further define relationships between NRs. PCC confirmed associations between groups of NRs, particularly for NRs in the ERα cluster, as well as a close relationship for the NR4A receptors, NURR1, NUR77 and NOR1 (Figure 4). Of particular note is the fact that expression of TLX, identified as an important variable in the Random Forest analysis, was negatively associated with ERα mRNA expression (PCC = −0.37, *p*-value = 1.97E-05). TLX expression was also negatively associated with clinical ERα (IHC) status (*p* = 5.5E-07; Table 2). However, evidence for association of DAX1 expression with ERα status (*p* = 2.9E-02) and ERα mRNA (PCC = −0.10, *p* = NS) was seen to be weak. Nor did examination of the microarray data indicate an association between ERα and DAX1. By contrast, the negative association between ERα and TLX was confirmed in the METABRIC (*p* = 5.5E-07) and GSE20685 (*p* = 1.3E-02) datasets. As ERα is a positive prognostic factor in breast cancer, TLX expression might be expected to be associated with poor outcome. Survival analyses showed that TLX expression in breast cancer is indeed associated with poor survival in the GSE20685 (HR = 1.77 (1.12 – 2.79), *p* = 0.0137) and METABRIC (HR = 1.32 (1.01 – 1.71), *p* = 0.041) series (Figure 5). Our analyses also showed that TLX is predominantly expressed in poor prognosis, ERα-negative breast cancers. Intriguingly, however, in these tumours, high expression of TLX in the absence of ERα is related to better survival, as seen in ERα-negative (HR = 0.69 (0.48 – 0.99), *p* = 0.048) and the PAM50 basal subtype (HR = 0.58 (0.37 – 0.93), *p* = 0.023).

### TLX regulates morphology, growth and invasive behaviour of triple-negative breast cancer cells

**Figure 6:**
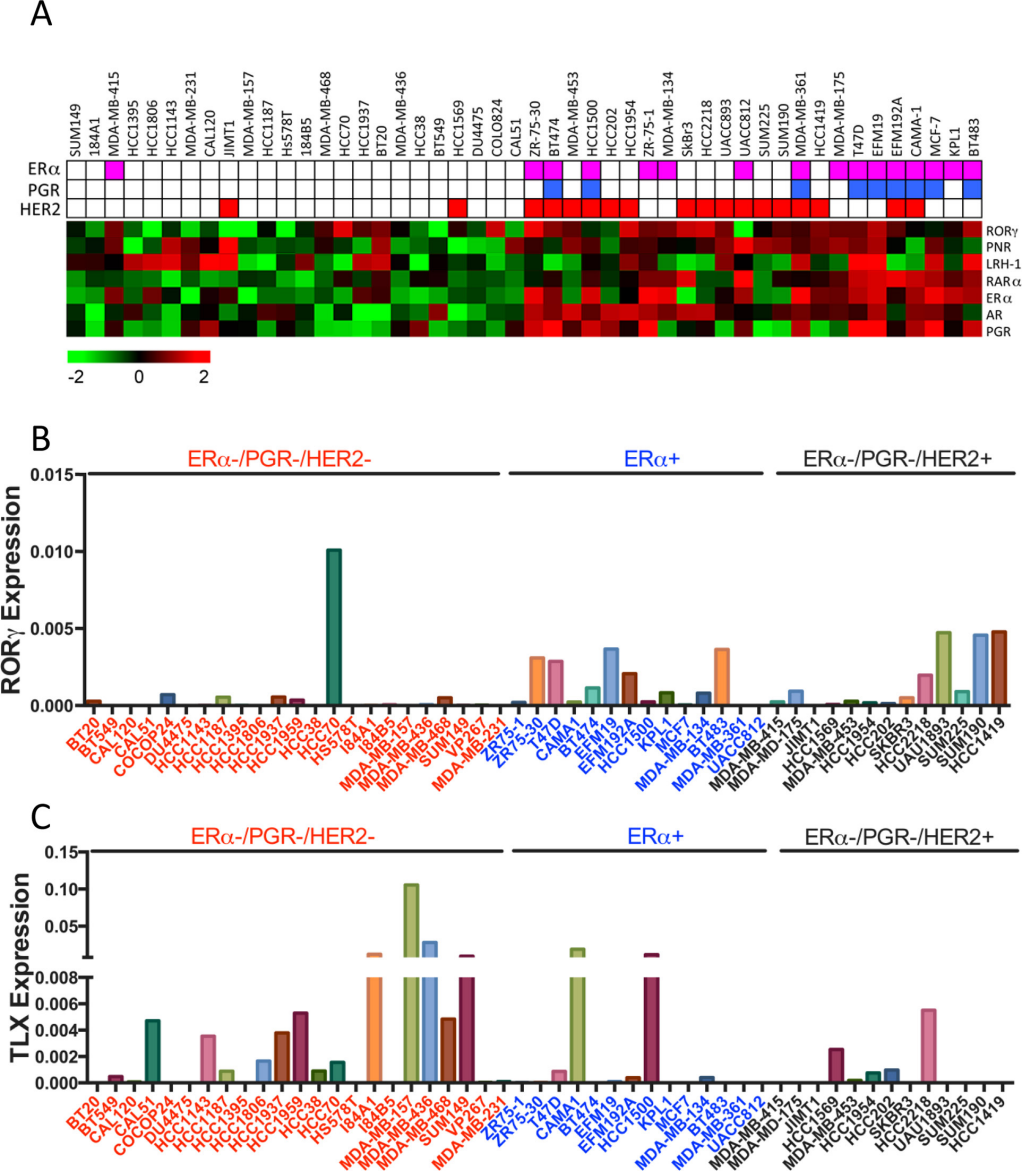
mRNA Expression of RORγ and TLX in Breast Cancer Cell Lines **A.** Heat map representation of NR expression in breast cancer cell lines is shown. The ERα, PGR and HER2 status of each cell line is also depicted. **B, C.** RORγ and TLX expression in breast cancer cell lines is shown relative to expression levels of GAPDH.

**Figure 7:**
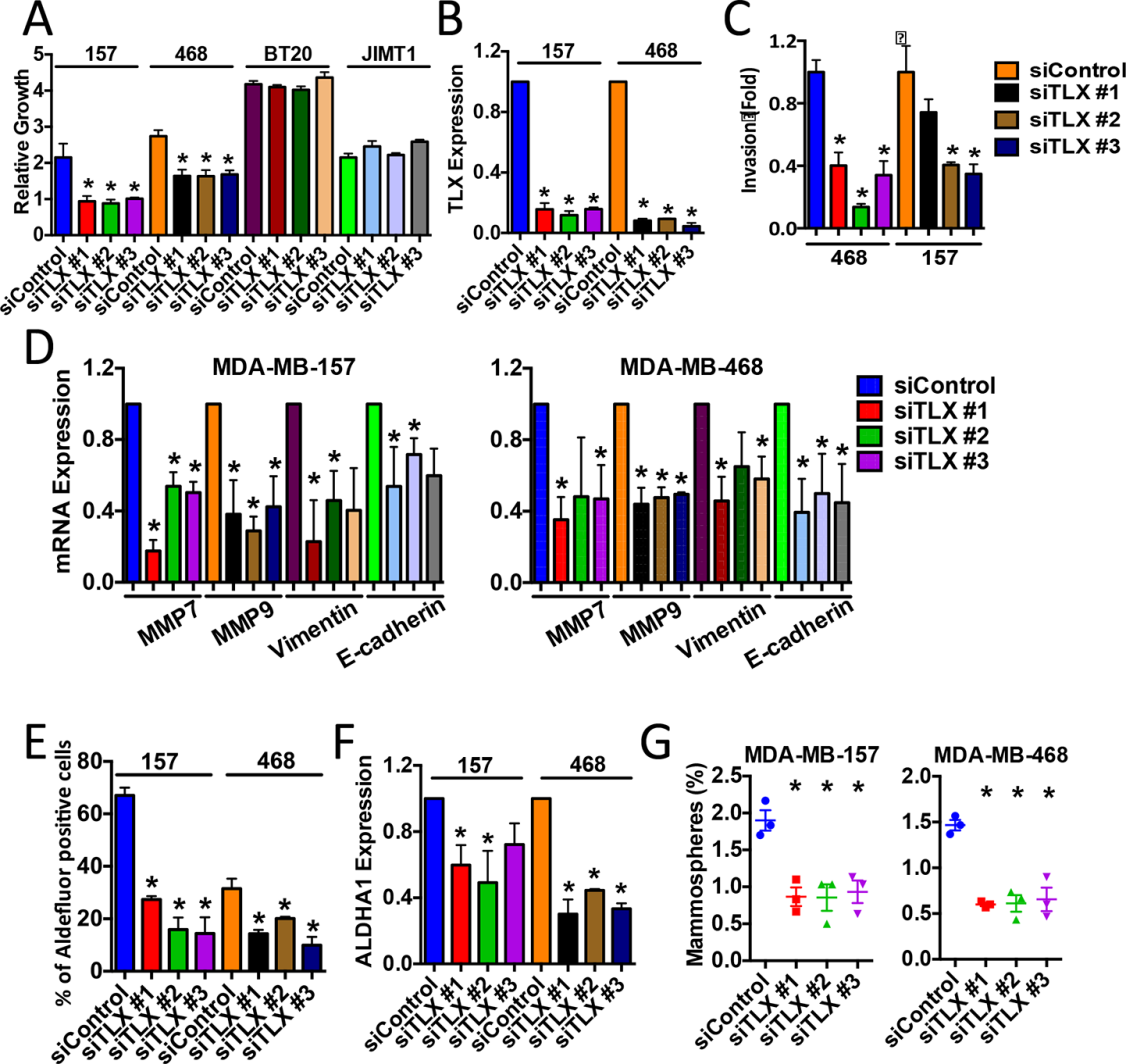
Targeted knockdown of TLX inhibits the growth of ERα-negative breast cancer cell lines MDA-MB-157 and MDA-MB-468 cells were transfected with a non-targeting siRNA (siControl), or with three independent siRNAs for TLX. Each experiment included three replicates and three independent experiments were carried out. Error bars show standard errors of the mean, asterisks represent *p* < 0.05, relative to the siControl. **A.** Cell number was assessed using the SRB assay 5 days following transfection. Growth is shown relative to the SRB values for day 0. **B, D, F.** Real-time RT-PCR was performed using RNA prepared from siRNA-transfected cells. mRNA expression was normalised to GAPDH expression. Bar charts represent mRNA levels compared to the siControl samples. **C.** Cells transfected with siRNAs were used in the transwell invasion assay. Bars represent number of invading cells. **E.** ALDH activity was determined using the Aldefluor assay. Bars show the percentage of Aldefluor-positive cells. **G.** Mammosphere forming efficiency (%) was calculated as the number of mammospheres formed in 10 days, divided by the original number of single cells seeded.

Our analyses highlight a potentially significant role for TLX in triple-negative and/or basal subtype breast cancer. Only 33% of ERα and/or HER2 positive breast cancer cell lines expressed TLX, compared with 60% of the triple-negative lines (Figure 6B). The role of TLX in triple-negative breast cancer was evaluated using MDA-MB-157 cells, which had the highest TLX expression in the breast cancer cell line panel. RNAi-mediated TLX knockdown clearly inhibited proliferation in MDA-MB-157 cells, an effect that was observed with three independent siRNAs (Figure 7A, B). TLX knockdown similarly inhibited the growth of another triple-negative cell line, MDA-MB-468, which expresses moderate levels of TLX. All three TLX siRNAs failed to affect the growth of two lines that do not express TLX (BT20, JIMT1), arguing against off-target effects being responsible for the growth effects due to these siRNAs observed in the TLX expressing lines.

**Figure 8:**
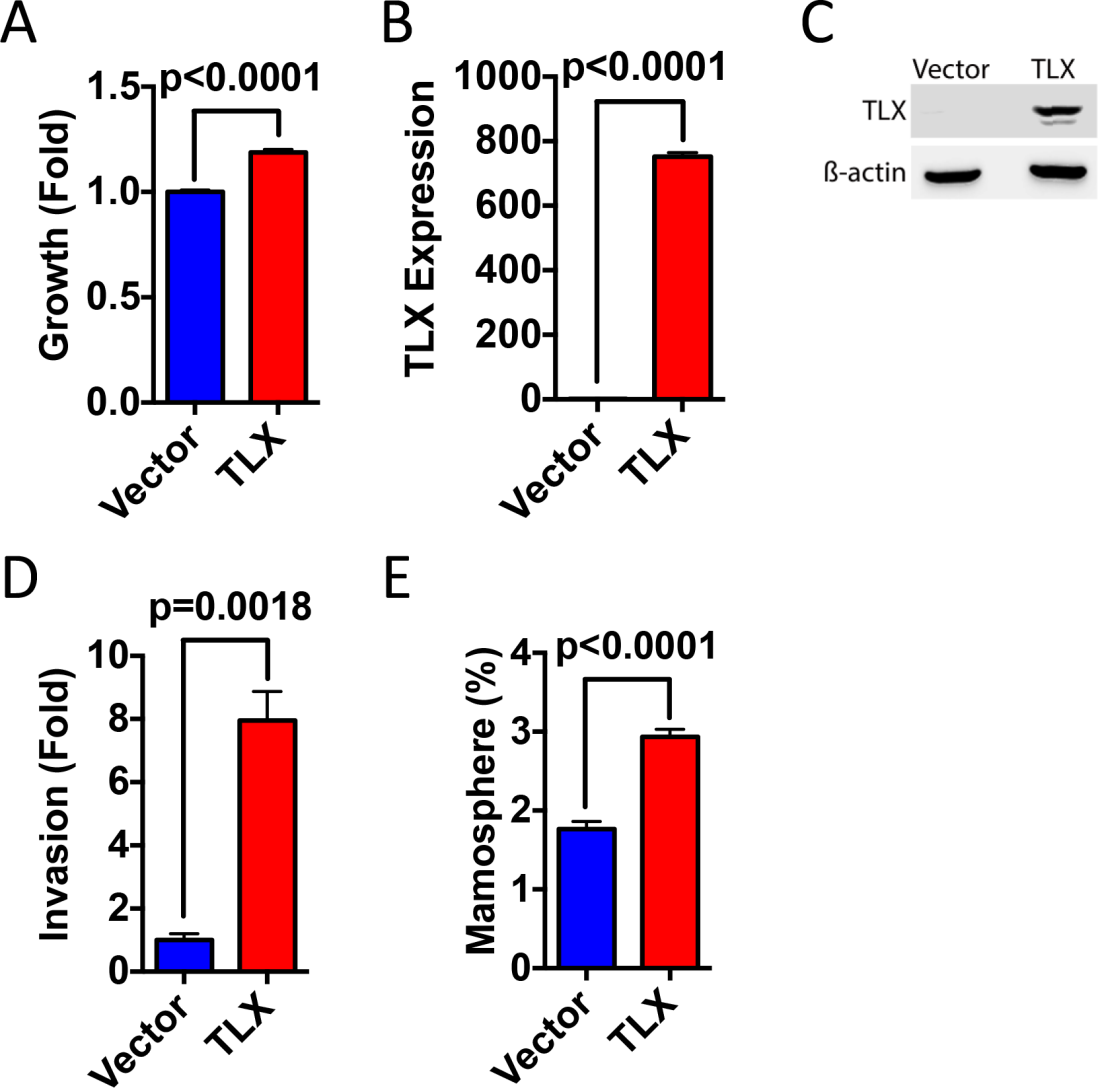
Ectopic expression of TLX promotes breast cancer cell growth, invasion and mammosphere formation MDA-MB-231 cells were transfected with TLX or with the vector control (pcDNA3). **A.** Cell number was assessed using the SRB assay 4 days following transfection. Growth is shown relative to the SRB values for the vector control (*n* = 3). **B.** Real-time RT-PCR was performed using RNA prepared from transfected MDA-MB-231 cells. mRNA expression was normalised to GAPDH expression. Bar charts represent mRNA levels compared to the vector control (*n* = 3). **C.** Protein lysates prepared from transfected cells were immunoblotted for TLX and β-actin. **D.** Cells transfected with TLX were used in the transwell invasion assay. Bars represent number of invading cells (*n* = 3). **E.** Mammosphere forming efficiency (%) was calculated as the number of mammospheres formed in 10 days, divided by the original number of single cells seeded (*n* = 6).

The invasive capacity of both lines was also markedly reduced following TLX silencing (Figure 7C), accompanied by significant inhibition in expression of epithelial-mesenchymal cell transition (EMT) markers such as MMP7, MMP9, vimentin and E-cadherin (Figure 7D). Since EMT can be associated with stem cell enrichment in breast cancer cells, we investigated the role of TLX on characteristics associated with breast cancer stem cells by investigating aldehyde dehydrogenase 1 (ALDH1) activity. TLX siRNA resulted in reduced expression of ALDHA1 and activity, determined using the Aldefluor assay (Figure 7E, F). The mammosphere assay provides an *in vitro* method for quantifying stem cell activity and self-renewal [25]. TLX knockdown resulted in a significant reduction in mammosphere forming efficiency in both cell lines (Figure 7G). Ectopic expression of TLX in MDA-MB-231 cells. In agreement with the siRNA results, TLX expression promoted growth, invasion and mammosphere formation in MDA-MB-231 cells (Figure 8), supporting the results of the TLX knockdown.

## DISCUSSION

Given the important gene regulatory roles for NRs in cancer and their drugability, we performed unsupervised hierarchical consensus clustering to analyse real-time, quantitative RT-PCR gene expression data for NR expression from 128 breast cancers. This allowed a broad separation of tumours into two prognostic groups, where univariate analysis suggested that ERα and PGR, together with other NRs acted to affect survival differences. To further investigate this, we have used unsupervised consensus clustering analysis to define associations between NR gene expression in breast cancer and have found evidence for three gene clusters.

NR gene cluster A comprised SHP, HNF4α and NR4A subfamily members. The NR4 subfamily members Nurr1, Nur77 and NOR1 were highly expressed in most samples, irrespective of tumour subtype. By contrast, SHP and HNF4α expression was low or absent in most tumours. Therefore, the co-clustering of these NRs is likely to be a reflection of a lack of differential expression across the range of tumours in our patient series. The high level expression of all three members of the NR4A receptors across the tumours is interesting, particularly in light of recent observations of increased expression of these NRs in breast cancer, compared with the normal breast [26]. NR4A receptors are important, albeit in a context-dependent manner, positive or negative regulators of cell survival and apoptosis [27]. Indeed, siRNA-mediated Nurr1 knockdown reduced growth of tumour xenografts [28], whereas Nur77 agonists inhibit MCF-7 cell proliferation and promote apoptosis [29]. However, another study has demonstrated that Nur77 over-expression does not affect proliferation or apoptosis in a number of breast cancer cell lines; rather the migratory potential of breast cancer cell lines, as well as that of the immortalized MCF-10a cell line, was diminished [30]. Given the elevated expression of NR4A receptors in the majority of breast cancers, greater consideration of the function of these NRs and their potential as therapeutic targets and biomarkers in breast cancer is clearly warranted.

Gene cluster B contained ERα and a group of NRs previously associated with ERα in breast cancer, including PGR, AR, RARα, LRH-1, PNR, as well as TRβ, RORβ, and RORγ. We have referred to cluster B as the “ERα Cluster” and note that a key feature of this is a functional association with the estrogen response, as seen for the activity for ERα, together with RARα, [10, 11], and more recently LRH-1, where we have found a functional co-operation between this NR and ERα through the use of common gene regulatory elements [12]. The association between ERα and RORγgene expression in breast cancer and breast cancer cell lines is novel and would require further characterisation.

Gene Cluster C contained NRs whose activation has been associated with the inhibition of breast cancer cell growth, including GR, RARβ, RARγ, RORα, PPARγ and VDR [16, 17], as well as ERβ, TR2 and TR4 whose role in breast cancer may lie, at least in part, with their action in reducing or inhibiting ERα activity [31–33]. These NRs were not differentially expressed in ERα/PGR/HER2 subgroups, with the exception of RARγ whose expression appeared to be lower in HER2-positive than in HER2-negative tumours, although statistical significance for association with HER2 status was not reached (*p* = 0.076). Other NRs in cluster C that have been implicated in breast cancer include ERRα, which is highly expressed in our samples. ERRα regulates breast cancer cell proliferation in *in vitro* and *in vivo* models and genetic deletion of ERRα delays tumour formation in a transgenic model of HER2-induced mammary tumourigenesis [34]. Transcriptomic studies have in particular highlighted the importance of ERRα and metabolism in breast cancer cells [35].

An important consideration is the fact that non-cancer components, particularly the stroma, as well as lymphocyte infiltration may influence NR expression profiles, although NR expression in stroma and/or lymphocyte infiltration, may be of some importance in breast cancer progression. For example, LRH-1 drives aromatase expression in cancer-associated stroma [36], while lymphocyte infiltration is associated with good prognosis [37], and response to chemotherapy [38] in ERα-negative disease. Two major RORγ isoforms have been described, with RORγt being highly expressed in lymphocytes. Our expression NR profiling used a real-time assay that would detect RORγ1, as well as the RORγt isoform. It is therefore possible that expression of the RORγ expression, presents expression in infiltrating lymphocytes, in addition to its expression in the cancer cells. In mitigation, expression profiling of NRs in diverse mouse tissues showed that SF-1 is one of 17 NRs that are expressed at moderate to high levels in tissues associated with the immune system (spleen, thymus) [39]. The near absence of SF-1 expression suggests that infiltrating lymphocytes only make up a minor component of the tumour cell population in our samples. Notwithstanding, we note that RORγ is expressed in many breast cancer cell lines and its expression is greater in ERα-positive than In ERα-negative cell lines, a relationship that is also observed in the tumour RNAs, as well as in microarray datasets. Further exploration of the functional importance of RORγ in breast cancer is therefore warranted.

Our analysis has also highlighted TLX as a gene of interest, since it is important in differentiating the two tumour clusters we have defined, where it is negatively associated with ERα mRNA expression and is associated with poor survival. Interestingly, TLX is one of a small number of genes expressed in breast cancer that predict for the presence of circulating tumour cells (CTC) [40]. Furthermore, tumours classified as CTC-positive in that study were more likely to be ERα-negative. Low level/absence of TLX expression in ERα-positive breast cancer and elevated expression in ERα-negative breast cancer may be due to the proposed different cellular origins of luminal and basal breast cancer sub-types, due to their origin in luminal and basal cells, respectively, or from different progenitor cells [41]. Interestingly, the most closely related NR to TLX is PNR, which promotes ERα expression in breast cancer cells through direct recruitment to the ERα gene promoter [19]. TLX and PNR both bind as monomers to very similar DNA motifs and given that TLX appears to function mainly as a transcriptional repressor [42], raising the intriguing possibility that TLX might repress ERα expression from the PNR binding site.

TLX regulates the proliferation and renewal potential of neural stem cells, where it acts as a transcriptional repressor through the recruitment of co-repressors, histone deacetylases (HDAC) and lysine-specific histone demethylase (LSD1) [43, 44], whilst a recent study defined a role for TLX in the regulation of neural stem cell senescence [45]. p53 is frequently lost in gliomas and TLX over-expression promoted glioma formation in p53 null mice [46]. There is, however, no evidence for TLX function in tissues other than the brain and NR profiling in a panel of mouse tissues (which did not include the mammary gland) showed little or no expression in tissues other than in the CNS [39], although TLX expression has been reported in cell lines representative of some cancer types [47].

We have found that siRNA mediated inhibition of TLX expression in breast cancer cell lines leads to an inhibition of cell growth, invasive potential and cancer stem cell properties, supporting the conclusion that TLX is important in breast cancer. In neural stem cells, TLX represses the expression of negative regulators of cell cycle progression and proliferation, including p21, p57 and PTEN (60), as well as stimulating the Wnt/β-catenin pathway through the direct activation of *Wnt7a* [48]. Our data demonstrate a functional role for TLX in breast cancer cells, although we did not observe de-repression of p21, p57 or PTEN expression (data not shown). Nor was expression of Wnt7A altered. This indicates that the action of TLX in breast cancer cells is mediated through target genes that are distinct from those implicated in neural stem cells. Other potentially relevant mechanisms of TLX function include the regulation of microRNAs, including the up-regulation of miR-9 [49], which has been implicated in breast cancer [50, 51]. Clearly, as a potential target, the mechanism by which TLX functions in ERα-negative breast cancer requires further investigation.

In summary, quantitative, real-time RT-PCR analysis of breast cancers revealed that the majority of NRs are expressed in breast cancer. Unsupervised hierarchical consensus clustering separated the tumours into clusters with prognostic significance. As expected, ERα and PGR were key variables in the prognostic groups. These analyses NRs whose expression profiles are indicative of their importance in different breast cancer subtypes. However, our results do not preclude the importance of other NRs, which are widely expressed across breast cancer subtypes, for example COUP-TFs, Nurr1/Nurr77/NOR1, RXRs, LXRs and PPARs. Indeed, a recent study mapping global binding sites for 24 nuclear receptors in the ERα-positive MCF-7 breast cancer cell line [52] provides evidence for the coordinate binding of many NRs to target regions throughout the genome, as exemplified by PPARδ, which as our results show is widely expressed across breast cancer subtypes. Our clustering analysis does nevertheless identify the orphan nuclear receptor TLX as an important variable in breast cancer, which is preferentially expressed in ERα-negative breast cancer where its importance is indicated by the prognostic significance of its expression and siRNA mediated growth inhibition and EMT. Secondly, we have uncovered a significant association between ERα and RORγ expression. Taken together, our profiling exercise describes two NRs whose function in breast cancer needs greater study.

## MATERIALS AND METHODS

### Human breast cancer samples

The patients presented with primary, operable breast cancer to the Dundee Cancer Centre between 1997 and 2012 and provided written, informed consent for research use of their tissues. Use of the clinical material and data was approved by the Tayside Tissue Bank under delegated authority from the Tayside Local Research Ethics Committee. ERα, PGR and HER2 immunohistochemical staining and scoring were carried out as described [53]. For HER2, all cancers scoring “equivocal” (2+) by IHC, were subjected to HER2 FISH analysis carried out using the PathVysion™ HER2 DNA probe kit (Vysis, Abbott Laboratories, Illinois, USA), with amplification of 2 fold or greater considered HER2 amplified. HER2 IHC positive (3+) cases, as well as FISH-positive HER2 IHC 2+ cases were scored as HER2-positive.

### Cell lines

Authenticated cell lines obtained from American Type Culture Collection were cultured according to ATCC recommendations for RNA preparation. Cell lines were authenticated using the short tandem repeat (STR) profiling service (LGC Standards, UK) immediately prior to initiation of studies.

### RNA extraction

Frozen breast tumour tissue (50 to 100 mg) was homogenized using TissueLyser (Qiagen) with stainless steel ball bearings (5 mm) in 0.7 ml of Lysis/Binding Buffer (Ambion) and total RNA was isolated according to the manufacturer's instructions. Removal of contaminating DNA from the RNA preparation was performed using DNA-free™ kit (Ambion/Life Technologies) following the manufacturer's protocol. The concentration and purity of total RNA was assessed using a NanoDrop™ 1000 spectrophotometer (Nanodrop Technologies, Wilmington, DE, USA).

### Reverse transcription

2.5 μg of total RNA was reverse converted to cDNA in a volume of 20 μl using RevertAid M-MuLV reverse transcriptase (Fermentas, York, UK), according to manufacturer's protocols. The reactions were carried out in the GeneAMP 9700 PCR machine (Applied Biosystems/Life Technologies, Paisley, UK) at 42°C for 1 hour, followed by 5 minutes at 95°C.

### TaqMan low-density array (TLDA)

384-well TLDA microfluidic cards were designed so as to include the Taqman real-time gene expression assays for 47 of the 48 human nuclear receptors that are included in the Taqman Human Nuclear Receptor Array (ABI cat. No.: 4379961). The Taqman assay for COUP-TF1 (NR2F1) was excluded from our TLDA array as the manufacturer has flagged this assay as amplifying COUP-TF2 (NR2F2), in addition to NR2F1. No other pre-designed Taqman assays exclusively amplifying COUP-TF1 were available from ABI at the time. Also included in our TLDA design was an assay for the glycerol-2-phosphate dehydrogenase (GAPDH) gene, as a control. The TLDA cards were purchased from Applied Biosystems (Life Technologies, Paisley, UK). For each tumour sample, cDNA was diluted 1:10 in ribonuclease-free water. A total volume of 100 μl reaction mixture, containing 50 μl of diluted cDNA and 50 μl of Taqman universal master mix was added to a TLDA fill reservoir. Each TLDA card was designed to assay 8 individual samples. The TLDA cards were then centrifuged twice at 1200 rpm for 2 minutes, sealed and run on an ABI 7900HT real time instrument (Life Technologies, Paisley, UK).

### Expression analysis of TLDA data set

TLDA card data were analysed using SDS2.2/RQ manager (ABI/Life Technologies). The quantification cycle threshold was kept constant and set at 0.15 across samples for data comparison, following manufacturer's instructions. The relative quantification to GAPDH expression was performed using DataAssist™ v3.0 (ABI/Life Technologies) and results exported to Microsoft excel for data processing. All values in the data set were log-2 transformed for further statistical analysis, as has been described [39].

### Statistical analysis

Statistical analyses were performed using R 2.15.1 software. Additional software packages (Consensus Cluster Plus, randomForest, corrplot, Hmisc, GMD, survival) were downloaded from the Comprehensive R Archive Network (CRAN).

Unsupervised consensus clustering was performed using R package ConsensusClusterPlus [18], using a median-centered expression matrix. The procedure was carried out with 1000 repetitions, 80% item resampling (pItem), a maximum evaluated number of groups (k) of 10 and use of an agglomerative hierarchical clustering algorithm with a distance metric given by one minus the Pearson correlation.

Random forest (RF) analysis was carried out using R package RandomForest [24]. The tumour cluster classification derived from consensus clustering served as a categorical factor for the RandomForest algorithm to generate a model using z-score transformed qRT-PCR dataset. The number of variables (NRs), randomly selected as candidates at each node was set to 7 and 5, 000, 10, 000, 20, 000 or 50, 000 trees were grown to reach tree numbers at which classification error reached stability. The default values were used for the remaining parameters throughout the analysis.

The consensus clustering heatmap was generated using the heatmap.3 code provided in the GMD package [54]. The dendrogram (clustering tree) for tumour and NR clustering were directly extracted from the consensus clustering analysis. For visualization, the expression data were normalized using median centred approach.

Pairwise Pearson Correlation Coefficient (PCC) analysis of NR in tumours was computed using Hmisc package. The resulting correlation coefficient and the matrix of *p*-value data were used as input to plot the correlation matrix graph using corrplot.

Breast cancer specific survival was estimated using R package survival [55] with the non-parametric product limit method (Kaplan-Meier). Continuous data were categorized into high expression or low expression, based on lower/upper quartile cut offs. Univariate Cox proportional hazards regression were used for examination of risk factors and given with corresponding hazard ratios (HR) and 95% confidence intervals. A *p*-value < 0.05 was considered as significant and all *p*-values were two tailed.

### Gene expression microarray data

GSE20685 was downloaded from Gene Expression Omnibus (GEO) [23]. The TCGA breast dataset was downloaded from The Cancer Genome Atlas Data Portal [22]. The METABRIC microarray data have been reported previously [21].

### RNA interference

BT20, JIMT1, MDA-MB-157 and MDA-MB-468 cells were cultured in Dulbecco's modified Eagle's medium (DMEM) containing 10% fetal calf serum (FCS). Cells were transfected with double-stranded RNA oligonucleotides using the Lipofectamine RNAiMax reverse transfection method (Invitrogen, UK), as described previously [12]. TLX siRNA #1 (s14200), #2 (s14201), #3 (s14202) and control siRNA (4390846) were purchased from Ambion. RNA was prepared 48 hours following transfection and real-time RT-PCR was performed, as described above. Real-time assays for TLX (Hs01128417_m1), ALDH1A1 (Hs00946916_m1), MMP7 (Hs01042796_m1), MMP9 (Hs00234579_m1), vimentin (Hs00185584_m1), E-cadherin (CDH1, Hs01023894_m1) and GAPDH (Hs99999905_m1) were purchased from ABI. Cell growth was determined using the sulphorhodamine B (SRB) assay, as described previously [12]. The transwell cell invasion assay, mammosphere culture and determination of ALDH activity were carried out as previously described [56].

### TLX transfection

MDA-MB-231 cells were cultured in Dulbecco's modified Eagle's medium (DMEM) containing 10% fetal calf serum (FCS). Cells were transfected with a FLAG-tagged TLX expression plasmid [57] using Fugene HD (Promega, UK), as described previously [12].

## SUPPLEMENTARY FIGURES


