# Supplementary material for: Expression profiling of nuclear receptors in breast cancer identifies TLX as a mediator of growth and invasion in triple-negative breast cancer
Source: Oncotarget. 2015 May 13;6(25):21685–703. doi: 10.18632/oncotarget.3942 (PMC4673296; doi:10.18632/oncotarget.3942)
Supplement: Supplementary file 1 [file oncotarget-06-21685-s001.pdf]

SUPPLEMENTARY FIGURES

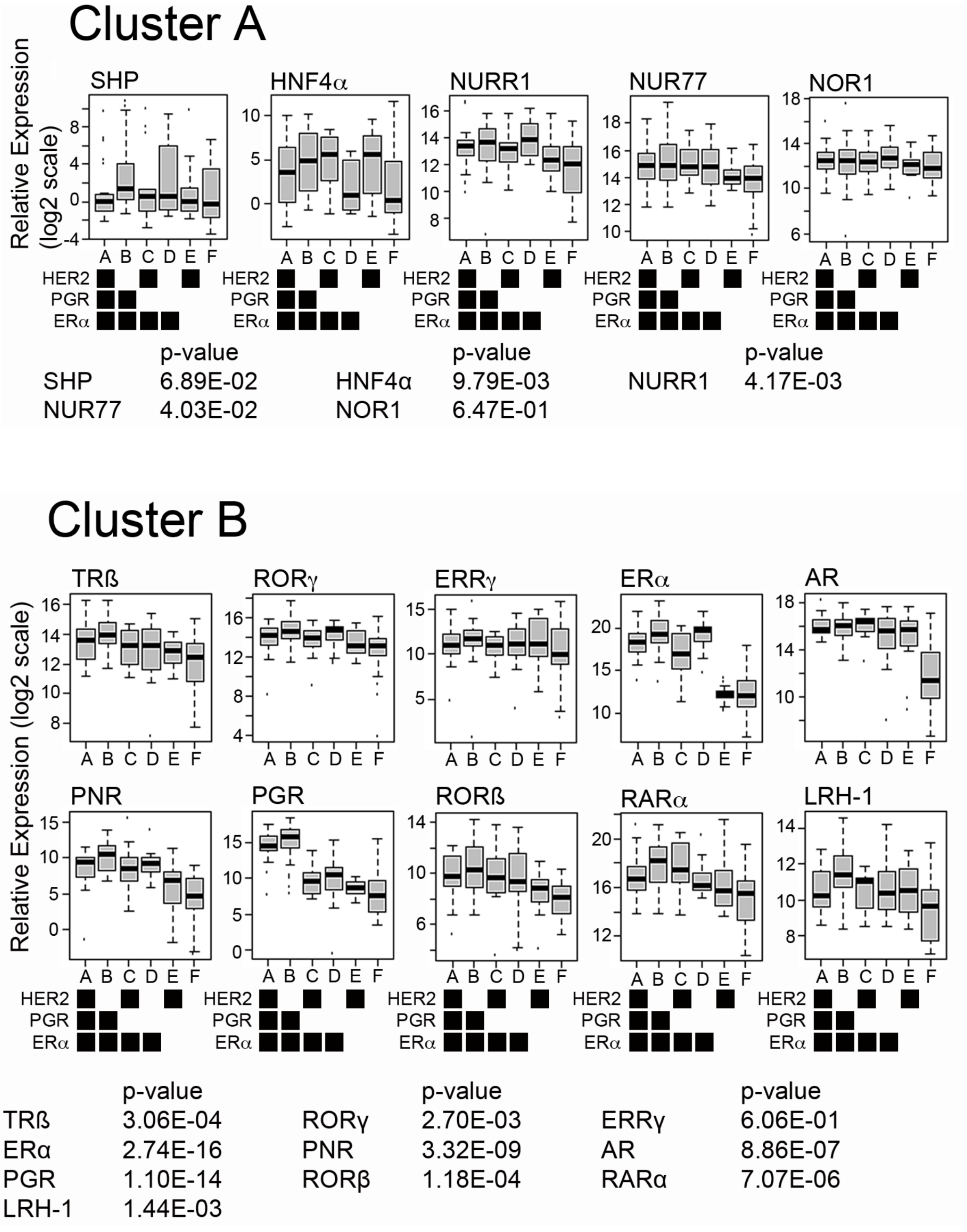

Supplementary Figure 1: Expression of nuclear receptors in different breast cancer subtypes. The expression of each NR in clusters A and B from the unsupervised hierarchical clustering analysis is shown in the form of a box whisker plot, for breast cancers classified on the basis of ERα, PGR and HER2 expression, the black boxes depicting positivity for each marker. The Kruskal Walls test derived *p*-values are shown.

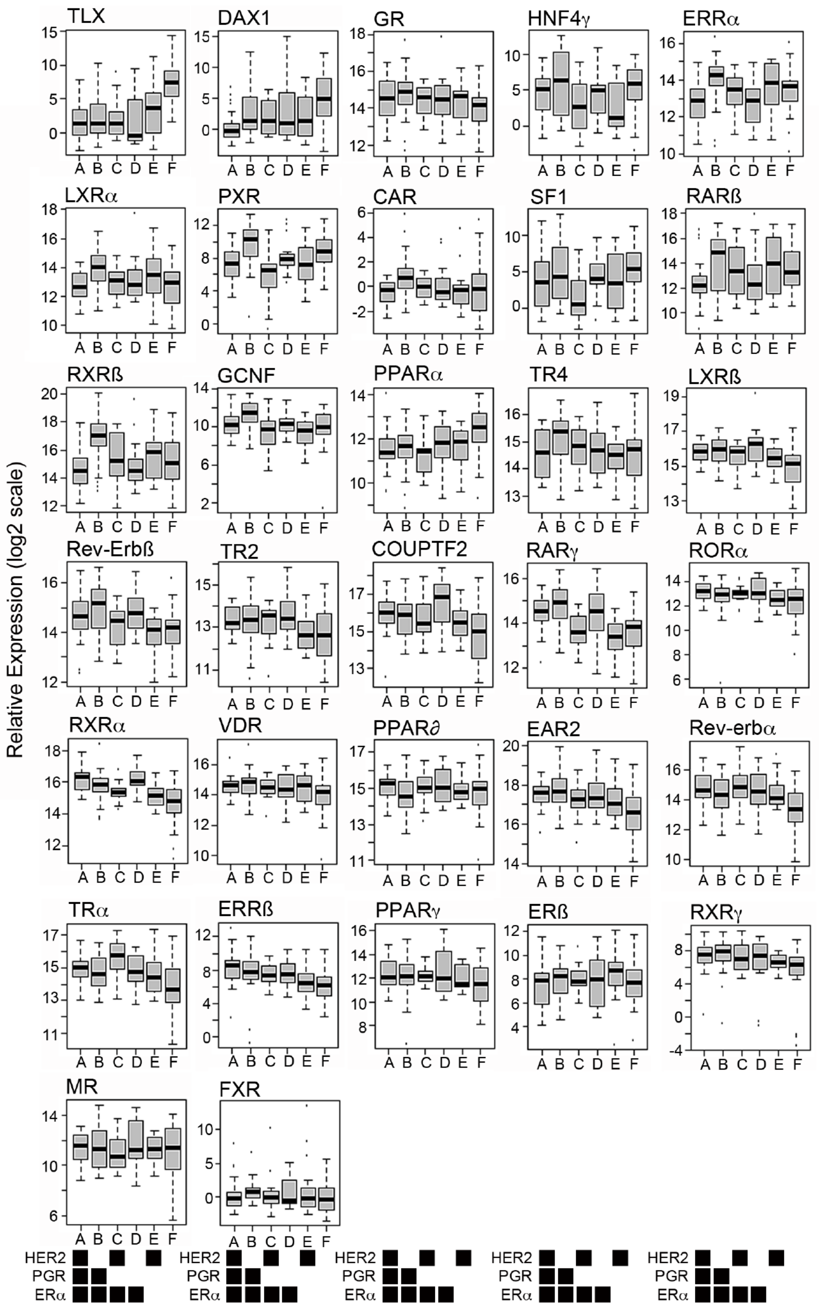

NR	p-value	NR	p-value	NR	p-value
TLX	6.72E-07	DAX1	8.67E-03	GR	1.68E-01
HNF4γ	2.26E-02	ERRα	7.27E-04	LXRα	1.91E-02
PXR	1.98E-04	CAR	3.62E-02	SF1	3.24E-02
RARβ	1.41E-01	RXRβ	1.02E-03	GCNF	4.42E-03
PPARα	9.00E-04	TR4	4.53E-02	LXRβ	5.33E-03
Rev-Erbβ	3.08E-03	TR2	4.81E-02	COUP-TF2	7.44E-03
RARγ	3.60E-06	RORα	1.97E-01	RXRα	1.57E-06
VDR	3.04E-01	PPARδ	4.35E-01	EAR2	5.48E-03
Rev-Erbα	6.84E-03	TRα	2.67E-03	ERRβ	1.89E-04
PPARγ	4.68E-01	ERß	5.71E-01	RXRγ	1.15E-02
FXR	1.72E-01				

Supplementary Figure 2: Expression of nuclear receptors in different breast cancer subtypes. The expression of each NR in cluster C from the unsupervised hierarchical clustering analysis is shown in the form of a box whisker plot, for breast cancers classified on the basis of ERα, PGR and HER2 expression, the black boxes depicting positivity for each marker. The Kruskal-Wallis test was used to determine significance.

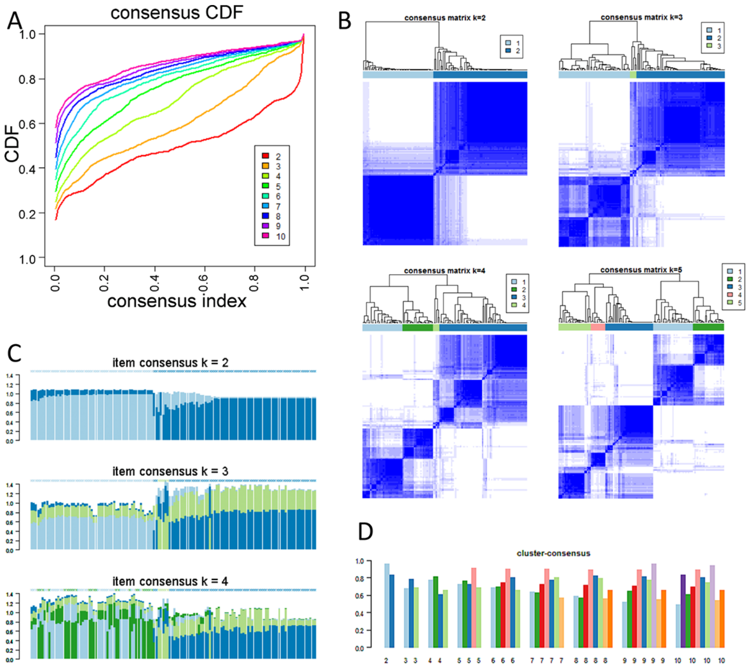

Supplementary Figure 3: Visual identification of robust tumour cluster numbers in consensus clustering analysis. **A.** Empirical cumulative distribution function plot displays consensus distributions for each k up to k = 10. **B.** Consensus matrix plots identify the cleanest cluster partition at k = 2. Tumour samples that always cluster together are coloured in dark blue and those that do not cluster together are in white. **C.** Item consensus (IC) plots display tumours as vertical bars and show a small subset of samples (in the middle) have mixed cluster association. IC plots at k ≥ 3 have more heterogeneous Item-consensus values which indicate that clustering of k ≥ 3 are less stable. **D.** Cluster consensus plot shows that a k ≥ 3 incurs low Cluster-consensus values.

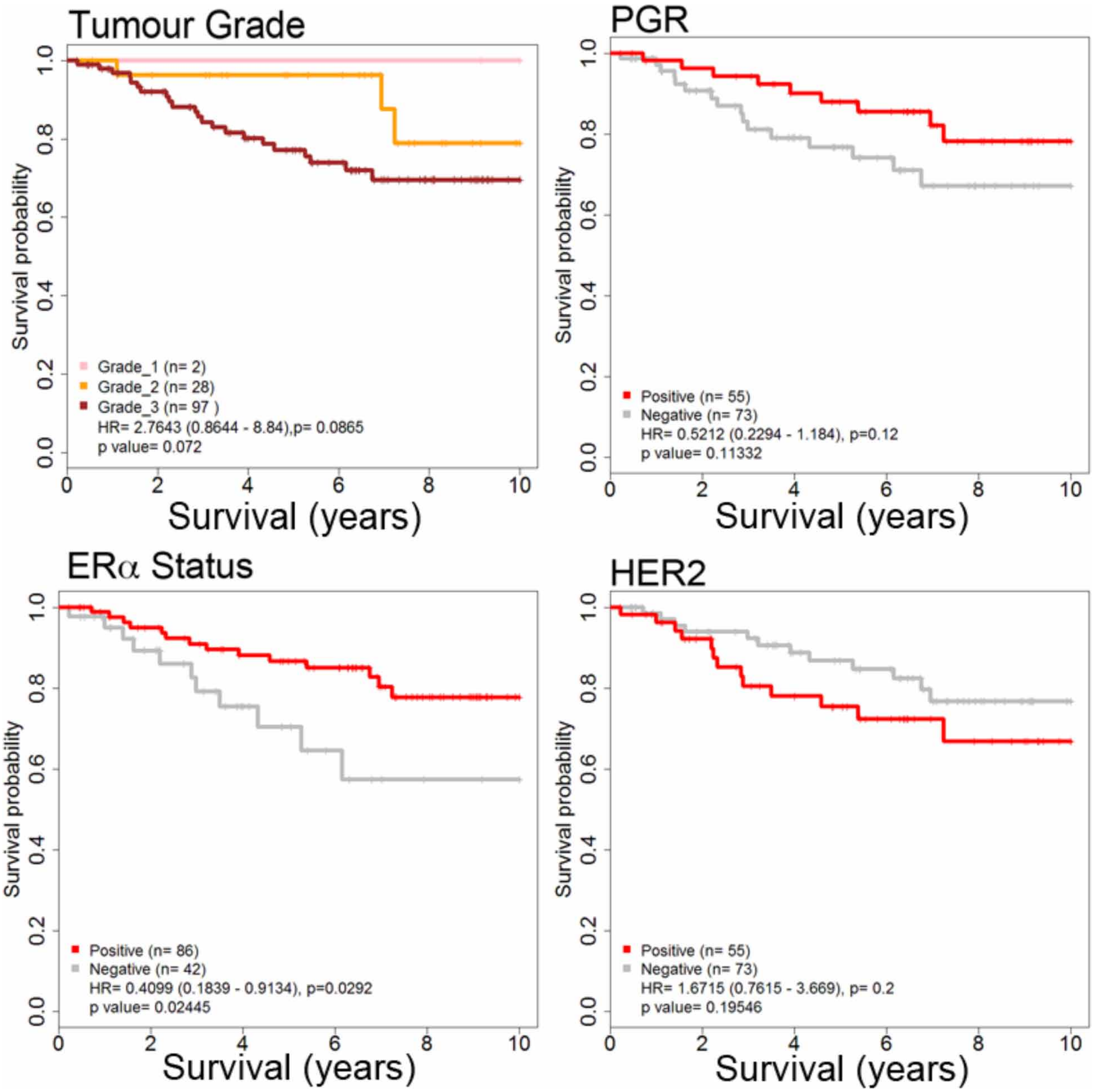

Supplementary Figure 4: Kaplan-Meier survival analysis. Crosses show censored samples. Univariate analysis was used to determine Hazard ratios (HR). HR values are given, together 95% confidence intervals.

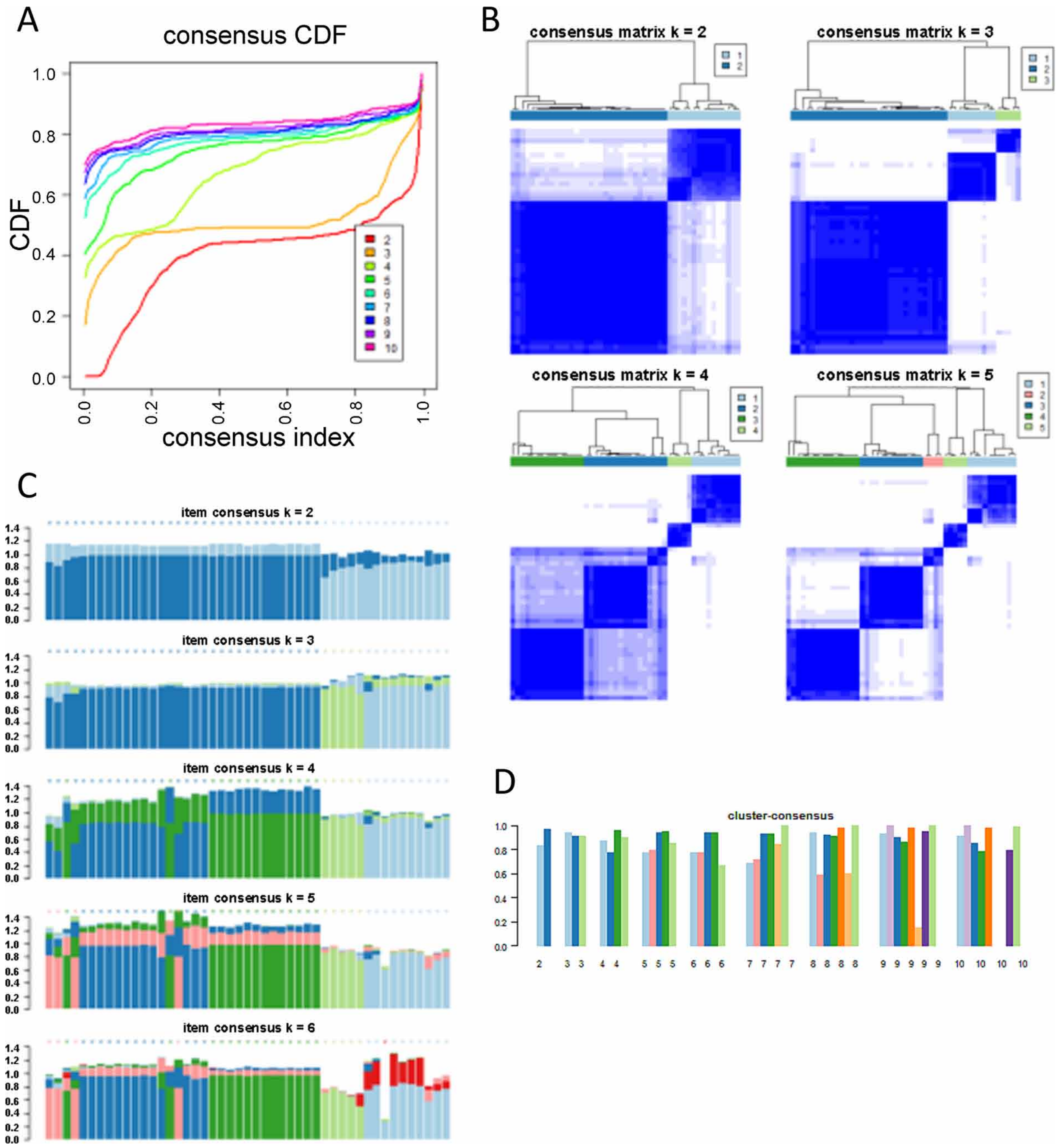

Supplementary Figure 5: Visual identification of robust cluster numbers for nuclear receptors (NRs) in consensus clustering analysis. **A.** Empirical cumulative distribution function plot displays consensus distributions for each k up to k = 10. **B.** Consensus matrix plots identify the cleanest cluster partition at k = 3. **C.** Item consensus plots display nuclear receptors as vertical bars and show that k = 3 has NRs with high consensus to their assigned cluster and low consensus to other clusters denoted by bars of mainly a single colour. **D.** Cluster consensus (CLC) plot at k = 3 shows high CLC between the clusters.
